# A new species and a new record of *Liparis* sect. *Decumbentes* (Malaxidinae, Orchidaceae) from Peru

**DOI:** 10.3897/phytokeys.146.47229

**Published:** 2020-05-08

**Authors:** Alexander Damián, Gerardo A. Salazar, Leyda Rimarachín

**Affiliations:** 1 Facultad de Ciencias Ambientales. Universidad Científica del Sur (Lima-Perú), Lima, Perú Universidad Científica del Sur Lima Peru; 2 Posgrado en Botánica Tropical, Facultad de Ciencias Biológicas, Universidad Nacional Mayor de San Marcos, Av. Venezuela, Cdra. 34 s/n, Lima, Perú Universidad Nacional Mayor de San Marcos Lima Peru; 3 Departamento de Botánica, Instituto de Biología, Universidad Nacional Autónoma de México, Apartado Postal 70-367, 04510 Mexico City, Mexico Universidad Nacional Autónoma de México México Mexico; 4 Universidad Politecnica de la Amazonia (UPA), Jr. Santa N° 47, Bagua Grande, Amazonas, Perú Universidad Politecnica de la Amazonia Amazonas Peru; 5 Area de Conservación Bosque Berlín, Bagua Grande, Amazonas, Perú Area de Conservación Bosque Berlín Amazonas Peru

**Keywords:** Andes, Amazonas, *Liparis brachystalix*, *Liparis sessilis*, neotropics, Andes, Amazonas, *Liparis brachystalix*, *Liparis sessilis*, neotropico

## Abstract

A new species of Liparis sect. Decumbentes, *Liparis sessilis* Damián, Salazar & Rimarachín, **sp. nov** is described and illustrated from Amazonas (Perú), including color photographs, a detailed comparison and an identification key to all the species of Liparis sect. Decumbentes. In addition, we report *Liparis brachystalix* Rchb.f. for the first time for the orchid flora of Peru, and select a lectotype for its synonym, *L. commelinoides* Schltr.

## Abstract (es)

Una nueva especie de Liparis sect. Decumbentes, *Liparis sessilis* Damián, Salazar & Rimarachín, **sp. nov** se describe e ilustra de Amazonas (Perú), incluyendo fotografías a color, una comparasion detallada y una clave de identificación de todas las especies de Liparis sect. Decumbentes. Ademas, reportamos *Liparis brachystalix* Rchb.f. por primera vez para la orquiflora del Perú, y seleccionamos un lectótipo para uno de sus sinónimos, *L. commelinoides* Schltr.

## Introduction

The genus *Liparis* Rich., in the traditional sense, is cosmopolitan and includes over 300 species (Cribb 2005). Although most authors have followed this broad concept (e.g. Ridley 1886; Schlechter 1921, Schweinfurth 1959; Bennett and Christenson 1993, 1995; Brako and Zarucchi 1993; Garay and Romero-González 1999; Ormerod 2012, 2016; Damián and Ormerod 2016), a molecular phylogenetic study by Cameron (2005) showed that thus delimited *Liparis* is grossly polyphyletic, and both its generic and infrageneric classification are pending revision.

The most recent proposal of a sectional classification of *Liparis* by Garay and Romero-González (1999) recognized 19 sections, but their monophyly has not been assessed. One of the sections proposed by Garay and Romero-González (1999), sect. Decumbentes, consisted of 4 South American species and was typified with *Liparis brachystalix*. This species was originally described from a plant collected in the surroundings of Quito (Reichenbach 1876) and was illustrated by a painting from a Colombian plant by Manuel Antonio Cortes (Mutis 1969: pl. 575; Fernandez 1992) executed during the Royal Botanical Expedition to the New Kingdom of Granada led by Jose Celestino Mutis. Cortes painted the plant more than 40 years before W. Jameson collected it in Ecuador but the results of the expedition remained unpublished until the 20^th^ century. Members of sect. Decumbentes are characterized by their decumbent or prorepent stems provided with secund or alternate leaves, and according to Garay and Romero González (1999) have an elongated column typical for the genus. However, most of the representatives included in this group (Table 1) have a short, straight column similar to that seen in *Crossoglossa* Dressler & Dodson (this last genus was treated by Garay and Romero-González as Liparis sect. Tipuloidea). *Liparis crispifolia* Rchb. f.is an exception, having a slender, arcuate column.

In Peru, sect. Decumbentes is represented by 3 species: *Liparis brachystalix*, *L. laticuneata* C. Schweinf. and a new species, in the following referred to as *L. sessilis* Damián, Salazar & Rimarachín. These are mostly restricted to the eastern slopes of the Andean Cordillera in the departments of Amazonas, Cusco, Pasco and Huancavelica where they grow as terrestrial (rarely epiphytic) plants between 2000–3000 m a.s.l. *Liparis crispifolia* has been cited for Peru by several authors, all of them following the dubious record of Schlechter (1921), who indicated “Cajamarca (?)” [sic]. Schweinfurth (1959) explicitly stated “*fide* Schlechter” in attributing this species to Peru, and subsequent works appear to have simply followed him (Brako and Zarucchi 1993; Zelenko and Bermúdez 2009; Goicochea et al. 2019). However, this taxon is endemic to the surroundings of Quito (Ecuador), where the type came from according to Reichenbach´s protologue, and recently re-collected in the same region (Dodson 1989; Dodson 2002; both reports misidentified as *L. nigrescens*).

During a field exploration conducted in the Private Conservation Area of the Berlin Forest (PCA BF), a protected area located in the northeast part of Peru, we collected a long epiphyte individual of *Liparis* sharing the distinctive vegetative features of sect. Decumbentes. After an extensive review of literature and herbaria we concluded that this specimen did not match any *Liparis* species described to date, and here we propose it as new. In the following, we provide a detailed description, a line illustration, color photographs and brief notes about the ecology of this specimen, and we compare it with other members of sect. Decumbentes.

## Materials and methods

A live flowering plant of the new species was collected on March 9, 2016 while conducting a floristic study in Bosque Berlin (Amazonas). The species was photographed *in situ* and also from ethanol preserved floral material using a Nikon D810 camera with Nikkor 60 mm lens. Herbarium specimen were prepared to be used as type material, and deposited at UFV and HUT (Acronyms following Thiers 2019). Descriptions and measurements were carried out under an Euromex SB-1903 and an AmScope SM-3TZ-54S-10M stereomicroscopes. The line illustration of the new species was prepared from alcohol-preserved material and digital photos. A total of 31 exsiccates of other members of Liparis sect. Decumbentes were compared for this work from the following herbaria: USM, MOL, HOXA, AMAZ, HUPCH, HSP, F, MO, HNOP, MEXU, QCE, QCNE and NY. We conducted a careful comparison of the new species with the protologues and type material of all species belonging to Liparis sect. decumbentes, as well as regional floras and checklists such as Schweinfurth (1959), Brako and Zarucchi (1993) and Ulloa Ulloa et al. (2004, 2017).

## Taxonomic treatment

### Liparis sessilis

Taxon classificationPlantaeAsparagalesOrchidaceae

Damián, Salazar & Rimarachín sp. nov.

9C973EBE-D0A5-5E4A-97FB-D05C43D3D8E9

urn:lsid:ipni.org:names:77209563-1

Figs 1A, E , 2

#### Type.

Peru. Amazonas; Bagua Grande, Bosque Berlín-“plot Higueron”, UTM 17 M 0786059, 9346365, 2300 m a.s.l. March 9, 2016, *L. Rimarachín LR 517* (holotype: UFV, isotype: HUT).

Similar to *Liparis brachystalix* Rchb.f. but differing in having sessile leaves (vs. distinctly petiolate) and ovate-elliptic labellum with truncate base (vs. obovate-oblong to pandurate labellum with cordate to sagittate base).

#### Description.

Terrestrial or epiphyte, long-creeping herb. ***Stem*** elongate, decumbent, laterally compressed, green, 2 mm in diameter, up to 35 cm. ***Leaves*** distichous, sessile, ovate, acute, with a narrow base clasping the stem, the margins undulate, 3–veined, 2.5–3.5 cm long and 1–1.5 cm wide. ***Inflorescence*** terminal, erect, racemose, producing many (up to 20) flowers in succession, peduncle terete in cross-section, provided with conspicuous glandular trichomes up to 6 mm long. ***Floral bracts*** pale greenish, narrowly triangular, margins undulate, 7–8 mm long and 2 mm wide. Ovary terete, with longitudinal keels, pale greenish, to 8 mm long including the pedicel. ***Flowers*** resupinate, widely spreading, sepals, petals and column pale greenish, labellum green, darker towards the center on the basal one-third. ***Dorsal sepal*** lanceolate, apex convex, broadly triangular, obtuse, margins revolute, 1–veined, 6–7.5 mm long and 1–1.5 mm wide. ***Lateral sepals*** lanceolate, slightly oblique, broadly rounded, margins revolute, 1–veined, 8 mm long, 2 mm wide. ***Petals*** linear, truncate with a rounded mucro, margins irregular, revolute, 1–veined, 7.5 mm long, 1 mm wide. ***Labellum*** ovate-elliptic, apex obtuse, upper lateral margins irregularly erose, 11–veined, 10–12 mm long, 4–5 mm wide, ecallose, with a squarish fovea above the base, medially with two low ridges converging up to three-quarters of the lamina. ***Column*** short, stout, 1.7 mm long; ***anther*** cucullate, 2–celled. ***Pollinia*** 2, obovate. ***Fruit*** unknown.

#### Etymology.

The specific epithet refers to the distinctive sessile leaves of the species.

#### Distribution and ecology.

This species inhabits the cloud forests around the river Utcubamba in the province of the same name in the district of Bagua Grande. This area is known as “El Higuerón” and is legally administered by the Rafael Cotrina family. This family, together with that of the third author, are conducting research and conservation programs about the yellow-tailed woolly monkey *Lagothrix flavicauda* Humbolldt and its habitat in the PCA BF. Flowers have been recorded in March and April.

*Liparis sessilis* inhabits the understory rich in mosses, rocks and old stems of *Anthurium* Schott and *Psychotria* L. species. Also, this species has been using *Palicourea* shrubs as its phorophyte and can reach up to 2 m above ground. The roots of *Liparis sessilis* are poorly developed and the stems turn white or brownish as the younger parts of the plant grow. According to recent observations (Rimarachín pers. obs.), the population of this species is small. Indeed, it has only been found in a degraded area and in two other zones of primary forest. The species has been propagated from cuttings and is currently being grown in the PCA BF.

#### Comments.

Among the four species belonging to Liparis sect. Decumbentes, *L. sessilis* is most similar to *L. brachystalix*, which differs in having petiolate leaves and different labellum morphology. *Liparis sessilis* is easily distinguished from other members of its section by the features indicated in the key and in Table 1.

**Figure 1.**
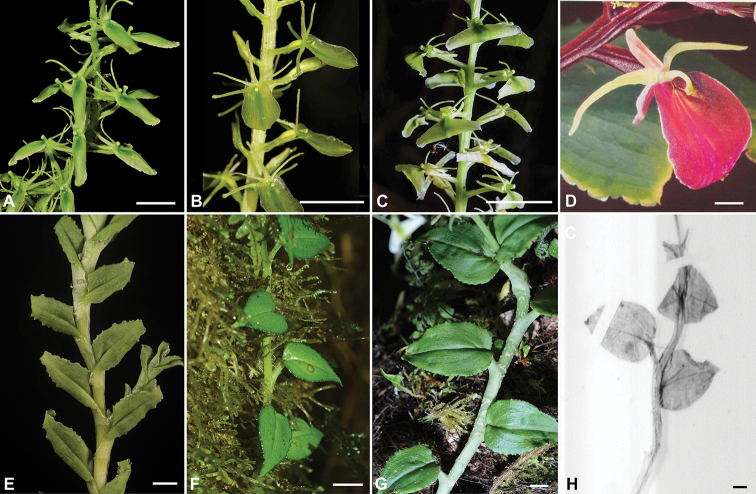
Flowers (**A–D**) and portions of stem with leaves (**E–H**) of the species of Liparis sect. Decumbentes**A, E***Liparis sessilis***B, F***Liparis brachystalix***C, G***Liparis laticuneata***D, H***Liparis crispifolia*. Photographs: **A** L. Rimarachín, **B** L. Egoavil, **C, E, G** A. Damián, **D** A. Hirtz, **F** G. Salazar. **H** E. Santiago. Scale bar: 1 cm.

**Figure 2.**
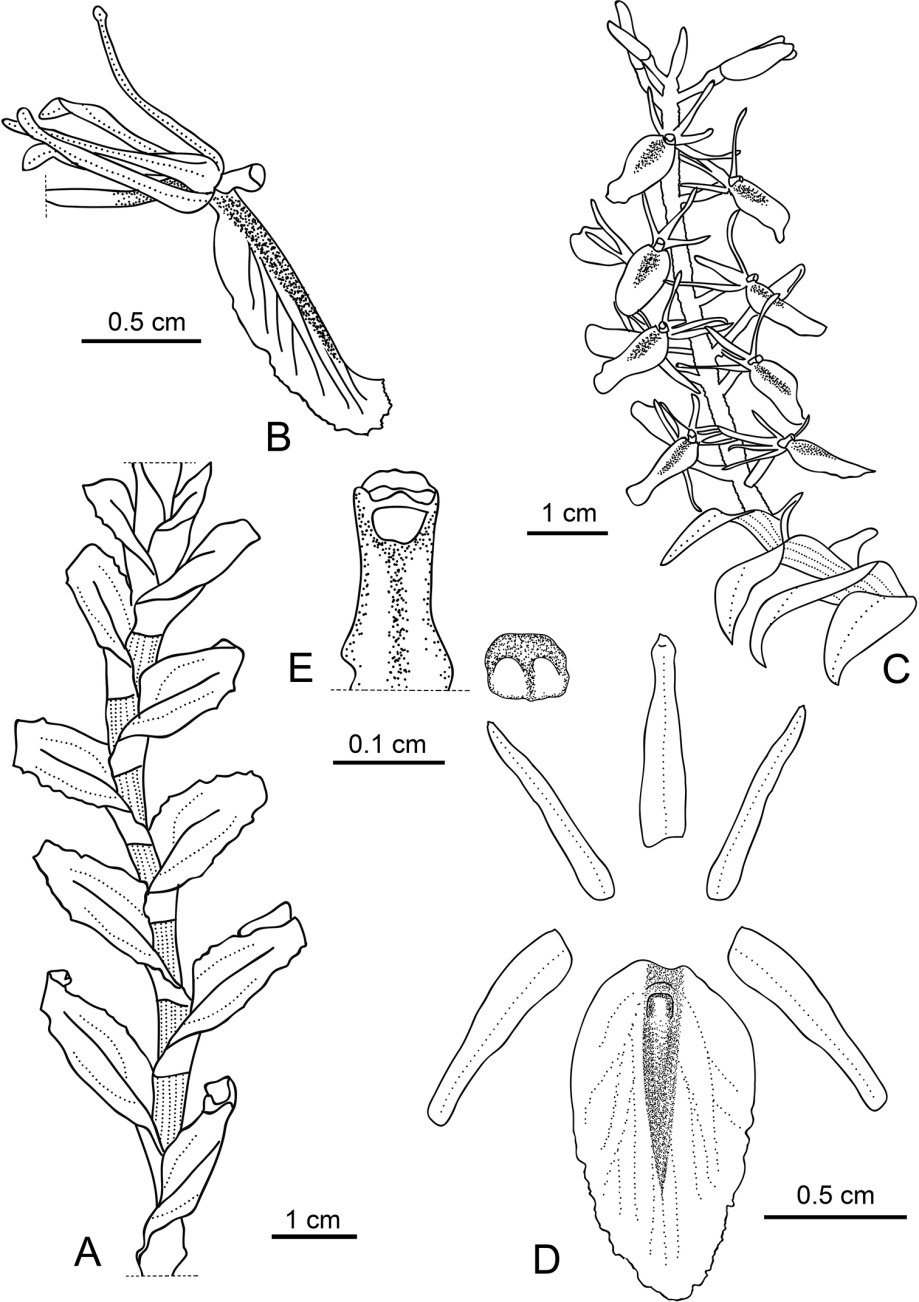
*Liparis sessilis* Damián, Salazar & Rimarachín **A** habit **B** lateral view of flower **C** inflorescence, **D** dissected perianth **E** column in ventral view with anther. Drawn by Alexander Damián from the type *L. Rimarachín LR517*.

**Table 1.** Features distinguishing the species of Liparis sect. Decumbentes.

Character	*L. brachystalix*	*L. crispifolia*	*L. laticuneata*	*L. sessilis*
**Leaf size (cm)**	1.6–3.7×0.9–2.2	5-6×3.7-4	2-5.5×2-3	2.5–3.5×1–1.5
**Leaf veins**	5–11	up to 11	up to 11	3–7
**Petiole length (cm)**	1.3–2.5	1.8–3	2–2.5	petiole absent
**Flower color**	Green with darker band along the center of the labellum	Greenish sepals and petals, labellum reddish purple	Green with darker band along the center of the labellum	Green with darker band along the center of the labellum
**Sepal size (mm)**	6–8×2	6–14×2	6×2	6–8×1–2
**Petal size (mm)**	6–9×1	5–14×6	6–7×2	5–7×1
**Labellum size (mm)**	7–8×3–7	16–40×10–36	7–8×10–13	10–12×4–5
**Labellum shape**	Obovate-oblong to pandurate	Oval to rhombic	Flabellate-cuneate	Ovate-elliptic
**Labellum base**	Strongly cordate to sagittate	Truncate	Shortly cuneate-truncate	Truncate,
**Labellum callus**	2–3 thickened veins	Base contracted to form a callus-like	V-shaped with an elliptic fovea	Squarish fovea above the base
**Column length (mm)**	2	4	1	2
**Column shape**	Straight or essentially so, stout	Arcuate, slender	Straight, stout	Straight, stout

### Liparis brachystalix

Taxon classificationPlantaeAsparagalesOrchidaceae

Rchb.f., Linnaea

FA1709BC-3DFF-5731-8A56-183D3116BC0A

**Figure 3.**
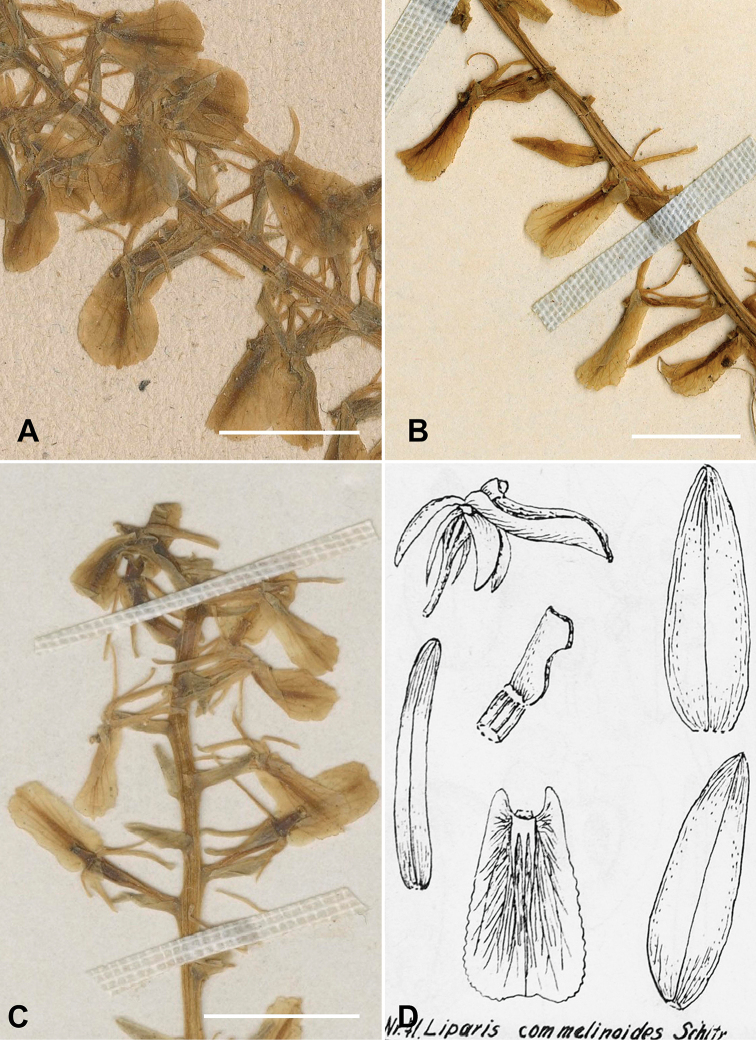
**A** holotype of *Liparis brachystalix* (*W. Jameson 448*, P) **B** holotype of *Liparis pothoides* (F.*C. Hehmann 8094*, AMES) **C** holotype of *Liparis fendleri* (*Fendler 1422*, PH) **D** Schlechter´s sketch of a flower from the holotype of *Liparis commelinoides*, published by Mansfeld (1930). Scale bar: 1 cm.

Liparis brachystalix Rchb.f., Linnaea 41: 43 (1876). Type: Ecuador, Pichincha, *Jameson*, *W. s.n*. (holotype US [drawing AMES!], isotypes AMES, GH, P (as *Jameson 448*).
Leptorkis brachystalix (Rchb.f.) Kuntze, Revis. Gen. Pl. 2: 671 (1891).
Liparis pothoides F.Lehm. &Kraenzl., Bot. Jahrb. Syst. 26: 478 (1899). Type: Colombia, Paramo de Guanacas, Central Andes of Popayan, *F. C. Lehmann 8094* (holotype K-photo, isotype AMES-photo).
Liparis commelinoides Schltr., Repert. Spec. Nov. Regni Veg. 14: 119 (1915). Type: Ecuador, Pichincha, *L. Sodiro 137* (holotype B, destroyed; Lectotype selected here: drawing of the holotype published by Mansfeld, 1930: no. 71. Fig. 3D).
Liparis fendleri Schltr. Repert. Spec. Nov. Regni Veg. Beih. 6: 32 (1919). Type: Venezuela, Prope Colonia Tovar, *A. Fendler 1422* (holotype AMES-photo!, isotypes GOET-photo!, AMES-photo!).

#### Remarks.

*Liparis brachystalix* is quite constant morphologically, having ovate, petiolate leaves with undulate margins, and labellum with a cordate base, apiculate apex, and a simple callus formed by thickened veins. Labellum shape varies slightly, being somewhat panduriform in the type of *L. pothoides* (Fig. 3B) to obovate-oblong in those of *L. fendleri*, *L. commelinoides* and *L. brachystalix* (Fig. 3A, C, D).

In Peru, *Liparis brachystalix* was collected in the 1960s in the cloud forests of Amazonas, but it was overlooked (Brako and Zarucchi 2993; Ulloa Ulloa et al. 2004, 2017). In a recent update of the checklist of Peruvian orchids of Zelenko and Bermudez (2009) published by Goicochea et. al. (2019), *L. brachystalix* was recorded for the first time in Peru. However, the record by Goicochea et al. (2019) did not indicate any vouchers supporting it. Therefore, herein we provide the first vouchered citation of *L. brachystalix* for this country. *Liparis brachystalix* is widespread along the eastern slope of the Peruvian Andes, inhabiting the cloud forest of Amazonas, Pasco, and Huancavelica at an altitude of 2000–2900 m.

#### Specimens examined.

Amazonas, Prov. Bongara, Dtto. Yambrasbamba, 1860–2000 m, 2-26 March 1967, *S. S. Tillet 673-304* (USM!); Pasco, Prov. Oxapampa, Dtto. Huancabamba, 10.25.45S 75.26.35W, 2870 m, 28 February 2009, *R. Vasquez*, *L. Valenzuela*, *J. Mateo & R. Rivera 35414* (USM!, HOXA!, HUT!, MOL!); Prov. Oxapampa, Dtto. Huancabamba, 10°26'35"S, 075°26'16"W, 2200.2500 m, 12 March 2006, *R. Vasquez et al. 31065* (HOXA!). Huancavelica, Prov. Tayacaja, Dtto. Tintay Puncu, Lihuapampa, Bosque nublado Usnopata-sector Vacayupana, 2900 m, 18 February 2015, *L. Egoavil s.n.* (photo!).

### Key to the species of Liparis sect. Decumbentes

**Table:** 

1	Leaves sessile; labellum convex towards the apex	***L. sessilis***
–	Leaves conspicuous petioles > 1 cm long; labellum concave or flat	**2**
2	Flowers green with a red-purple labellum; sepals and petals > 1 cm long; labellum ovate-rhombic, ecallose; column slender and arcuate	***L. crispifolia***
–	Flowers entirely green with a darker green longitudinal band on the labellum; sepals and petals < 1 cm long; labellum obovate-oblong or flabellate-cuneate, with a distinct callus; column stout and straight or essentially so	**3**
3	Labellum broader than long, shortly cuneate at the base and abruptly expanded above, recurved; column straight	***L. laticuneata***
–	Labellum longer than broad, cordate at base and not abruptly expanded above, flat; column curved or straight	***L. brachystalix***

## Supplementary Material

XML Treatment for Liparis sessilis

XML Treatment for Liparis brachystalix

## References

[B1] Bennett DE Christenson EA (1993) Icones Orchidacearum Peruviarum, 200 pp.

[B2] Bennett DE Christenson EA (1995) Icones Orchidacearum Peruviarum, 200–400 pp.

[B3] Brako L Zarucchi JL (1993) Catalogue of the flowering plants and Gymnosperms of Peru. Monographs in Systematic Botany from the Missouri Botanical Garden 45: 1–1286.

[B4] Cameron KM (2005) Leave it to the leaves: A molecular phylogenetic study of Malaxideae (Epidendroideae, Orchidaceae). American Journal of Botany 92(6): 1025–1032. 10.3732/ajb.92.6.1025 21652487

[B5] Cribb P (2005) *Liparis*. In: Pridgeon AM Cribb PJ Chase MW Rasmussen FN (Eds) Genera Orchidacearum (Vol.4). Epidendroideae Part 1. Oxford University Press, Oxford, 465–471.

[B6] Damián A Ormerod P (2016) *Liparis aphylla* (Malaxideae, Orchidaceae), a new leafless record from Peru. PhytoKeys 61: 27–35. 10.3897/phytokeys.61.7420 PMC4816979 27081347

[B7] Dodson CH (1989) *Liparis nigrescens* Schltr. In: Dodson CH (Eds) Orchids of Ecuador, Icones Plantraum Tropicarum series 2, fascicle 6. Missouri Botanical Garden, St. Louis, 512 pp.

[B8] Dodson CH (2002) Native Ecuadorian orchids 3. *Lepanthopsis*-*Oliveriana*. Dodson Trust, 433–651.

[B9] Fernandez PB (1992) Listado completo de láminas. In: Villegas Lunwerg (Eds) Mutis y la Real Expedición Botánica del Nuevo Reino de Granada.Comisión Nacional Quinto Centenario, Barcelona, 49–158.

[B10] Garay LA Romero-González GA (1999) Schedulae Orchidum II. Harvard Papers in Botany 4(2): 475–488.

[B11] Goicochea A Gutiérrez A Ruiz A Salas M (2019) Orquideas de Perú: Relación de Especies y sus Sinónimos. G&G Corporation S.A.C., 288 pp.

[B12] Mansfeld R (1930) Blütenanalysenneur Orchídeen von R. Schlechter. I. Südameríkanische Orchideen. Repertorium Specierum Novarum Regni Vegetabilis. Beihefte, 60 pp.

[B13] Mutis JC (1969) Microspermae (Orchidaceae II) Flora de la Real Expedicion Botanica del Nuevo Reino de Granada. Ediciones Cultura Hispanica, Madrid, 139 pp.

[B14] Ormerod P (2012) Notes on Liparis Section Ramosae (Orchidaceae: Malaxidae). Harvard Papers in Botany 17(1): 169–177. 10.3100/025.017.0118

[B15] Ormerod P (2016) Neotropical Orchid Miscellanea. Harvard Papers in Botany 21(2): 231–245. 10.3100/hpib.v21iss2.2016.n8

[B16] Reichenbach HG (1876) OrchideaeRoezlianae novae seucriticae. Linnaea 43: 1–98.

[B17] Ridley HN (1886) A monograph of the genus *Liparis*. The Journal of the Linnean Society (Botany) 22(145): 244–297. 10.1111/j.1095-8339.1886.tb00468.x

[B18] Schlechter R (1921) Die orchideenfloren der sudamerikanisehen Kordillerenstaaren, IV. Peru. Repertorium Specierum Novarum Regni Vegetabilis. Beihefte 9: 1–182.

[B19] Schweinfurth C (1959) Orchids of Peru. Fieldiana. Botany 30(2): 373–38.

[B20] Thiers B (2019) Index Herbariorum: A global directory of public herbaria and associated staff. New York Botanical Garden’s Virtual Herbarium. http://sweetgum.nybg.org/ih/

[B21] Ulloa Ulloa C Zarucchi JL León B (2004) Diez años de adiciones a la flora del Perú. Arnaldoa (edición especial): 7–242. 10.5962/bhl.title.63538

[B22] Ulloa Ulloa C Acevedo-Rodríguez P Beck S Belgrano MJ Bernal R Berry PE Brako L Celis M Davidse G Forzza RC Gradstein SR Hokche O León B León-Yánez S Magill RE Neill DA Nee M Raven PH Stimme H Strong MT Villaseñor JL Zarucchi JL Zuloaga FO Jørgensen PM (2017) An integrated assessment of the vascular plant species of the Americas. Science 358(6370): 1614–1617. 10.1126/science.aao0398 29269477

[B23] Zelenko H Bermudez P (2009) Orchid Species of Peru. Zai Publications. Quito, 407 pp.

